# The presence of hydrogenotrophic methanogens in the inoculum improves methane gas production in microbial electrolysis cells

**DOI:** 10.3389/fmicb.2014.00778

**Published:** 2015-01-15

**Authors:** Michael Siegert, Xiu-Fen Li, Matthew D. Yates, Bruce E. Logan

**Affiliations:** ^1^Department of Civil and Environmental Engineering, Penn State UniversityUniversity Park, PA, USA; ^2^School of Environmental and Civil Engineering, Jiangnan UniversityWuxi, China

**Keywords:** electromethanogenesis, biocathode, power-to-gas, microbially influenced corrosion, bog sediment, *Geobacter*, *Methanobacterium*, *Methanobrevibacter*

## Abstract

High current densities in microbial electrolysis cells (MECs) result from the predominance of various *Geobacter* species on the anode, but it is not known if archaeal communities similarly converge to one specific genus. MECs were examined here on the basis of maximum methane production and current density relative to the inoculum community structure. We used anaerobic digester (AD) sludge dominated by acetoclastic *Methanosaeta*, and an anaerobic bog sediment where hydrogenotrophic methanogens were detected. Inoculation using solids to medium ratio of 25% (w/v) resulted in the highest methane production rates (0.27 mL mL^−1^ cm^−2^, gas volume normalized by liquid volume and cathode projected area) and highest peak current densities (0.5 mA cm^−2^) for the bog sample. Methane production was independent of solid to medium ratio when AD sludge was used as the inoculum. 16S rRNA gene community analysis using pyrosequencing and quantitative PCR confirmed the convergence of *Archaea* to *Methanobacterium* and *Methanobrevibacter*, and of *Bacteria* to *Geobacter*, despite their absence in AD sludge. Combined with other studies, these findings suggest that *Archaea* of the hydrogenotrophic genera *Methanobacterium* and *Methanobrevibacter* are the most important microorganisms for methane production in MECs and that their presence in the inoculum improves the performance.

## Introduction

In a microbial electrolysis cell (MEC), the voltage generated by bacteria degrading organic matter on the anode can result in electrical current generation and hydrogen production at the cathode when additional power is added to the system (Liu et al., 2005; Rozendal et al., 2008; Logan and Rabaey, 2012). Hydrogenotrophic methanogens in the reactor can further convert hydrogen gas to methane gas by reducing CO_2_ (Clauwaert and Verstraete, 2009; Villano et al., 2011). It is also now known that methanogens can directly catalyze the release of electrons on the cathode to make methane via electromethanogenesis (Cheng et al., 2009; Villano et al., 2010; Lohner et al., 2014), circumventing the dependence on hydrogen gas. The production of methane rather than hydrogen gas may be desirable as methane can readily be used in the existing natural gas infrastructure. Furthermore, microbiological methane produced from CO_2_ and hydrogen gas is a renewable biofuel that can be stored, transported and converted to syngas. Biological conversion of hydrogen gas to methane can occur on the anode or the cathode in single-chamber MECs due to the release of the hydrogen gas into solution (Sasaki et al., 2011). Two-chamber MECs have a membrane between the electrodes to avoid hydrogen gas crossover from the cathode to the anode, but the use of a membrane can lead to pH changes in the system that reduce performance primarily due to a low anode pH (Rozendal et al., 2006; Kim et al., 2007). In single-chamber systems there is no membrane, and in general they produce higher current densities than two-chambered MECs (Call and Logan, 2008). Single-chamber MECs are simple in design, easier to construct, and they are often used for screening experiments to evaluate biodegradability of different wastewaters (Call and Logan, 2011; Ren et al., 2013).

The microorganisms that develop on the anode in MECs are well understood, particularly when acetate is used as the fuel. Most exoelectrogenic biofilms in MECs that produce high current densities consist predominantly of microorganisms most similar to various *Geobacter* species, and most often *Geobacter sulfurreducens* (Chae et al., 2008; Torres et al., 2009; Yates et al., 2012). Microbial fuel cells (MFCs) and MECs inoculated with a wide diversity of inocula, from both natural freshwater environments and engineered reactors (e.g., wastewater treatment plants), typically converge to communities containing predominantly *G. sulfurreducens* (Holmes et al., 2004; Yates et al., 2012). Both acetate and hydrogen can be used by *G. sulfurreducens*, although it has been shown that dissolved hydrogen gas can reduce acetate oxidation under iron reducing conditions (Brown et al., 2005). Based on this observation with iron as the terminal electron acceptor, it is likely that the utilization of hydrogen gas by microorganisms on the anode could reduce acetate oxidation by bacteria such as *G. sulfurreducens*. The use of hydrogen gas by the anodic biofilm results in high rates of hydrogen gas recycling, where hydrogen from the cathode is used to produce current at the anode (Rozendal et al., 2008; Lee et al., 2009; Tartakovsky et al., 2009). Hydrogen gas recycling is not desirable as electrical power is wasted by cycling hydrogen between the electrodes with no net gas production (Wang et al., 2009; Rader and Logan, 2010). The conversion of hydrogen to methane eliminates hydrogen gas recycling as there is no further conversion of methane to another chemical product.

The methanogenic microorganisms that develop on cathodes in single-chamber MECs are not well studied, particularly in systems where methane is the predominant gas recovered from the reactor. It seems likely that hydrogenotrophic methanogens would be most effective when using hydrogen during growth on the cathode, and electrotrophic methanogens must necessarily attach to the cathode. When acetate is used as the fuel, methane can also be produced through acetoclastic methanogenesis. Therefore, methanogens can produce methane on both the anode and cathode using either acetate or hydrogen gas. The first report of electromethanogenesis identified *Methanobacterium palustre* as the primary methanogen on a biocathode maintained at a set potentials ranging from −0.5 to −1.0 V vs. a standard hydrogen electrode (Cheng et al., 2009). Since then, primarily unknown *Methanobacterium* species have been found on methanogenic biocathodes (Sasaki et al., 2011; Van Eerten-Jansen et al., 2013; Jiang et al., 2014). Other methanogens identified to be present, but less abundant, on the cathodes and anodes of MECs and microbial fuel cells (MFCs) include *Methanobrevibacter, Methanocorpusculum, Methanosarcina*, and *Methanoculleus* species (Sasaki et al., 2011; Shehab et al., 2013; Jiang et al., 2014). All these genera are exclusively hydrogenotrophic methanogens (except *Methanosarcina* which uses acetate as well), despite the use of acetate as the fuel in these systems. Different communities of *Archaea* can develop when the operational conditions of the reactor are changed. For example, in MECs fed with waste activated sludge, air was used to try to inhibit the growth of methanogens on the anodes, and members of *Methanomicrobiales, Methanosarcinaceae*, and *Methanosaetaceae* prevailed while only low numbers of *Methanobacteriales* were found (Lu et al., 2012). On anodes of open circuit MFCs fed acetate, *Methanocorpusculum* species predominanted (Shehab et al., 2013). When ethanol was used as a substrate in two-chamber MECs, the predominant anodic genus was *Methanobrevibacter* (Parameswaran et al., 2010).

In order to better understand the methanogenic communities that are responsible for methane production in acetate-fed MECs operated with current generation, we examined microbial communities and gas production rates in MECs using two different inoculum sources: sediments from a natural bog; and anaerobic digester (AD) sludge from a local wastewater treatment plant. While acetate is a major precursor for methanogenesis in various freshwater sediments (Thebrath et al., 1993; Zepp Falz et al., 1999; Chan et al., 2002), although in some cases no acetoclastic methanogens are detected (Nüsslein et al., 2001), bogs in the local area of our laboratory have been found to comprise a greater variety of different methanogens among which hydrogenotrophic methanogens were found as well (Steinberg and Regan, 2008). The use of a bog sample in an MFC was found to result in more rapid acclimation of the anode for current generation, reaching maximum power production over fewer cycles than MFCs inoculated with domestic wastewater (Yates et al., 2012). AD sludge at most domestic wastewater treatment plants predominantly contains acetoclastic methanogens. The sludge has a relatively high organic load compared to the more predominantly inorganic composition of bog sediments. Therefore, we inoculated reactors with either bog sediment or AD sludge at different solids to medium ratios (0.01, 0.1, 1, 10, and 25%; w/v). We examined the effect of inoculum size on the subsequent methane production rates and current densities, and the extent of hydrogen gas recycling by Coulombic efficiencies (Coulombs produced versus those in the added substrate). To characterize microbial community in the electrode biofilms, we used 16S rRNA gene pyrosequencing to determine diversity and composition of the inocula and quantitative PCR (qPCR) to quantify archaeal and bacterial 16S rRNA genes.

## Materials and methods

### MEC reactors and operation

Mini-MECs were prepared as described by Call and Logan (2011) using 5 mL clear glass serum bottles (Wheaton, Millville, NJ, USA). Both electrodes were graphite plates 0.32 cm thick, 1.5 cm long, and 1 cm wide, with a total of 1.5 cm^2^ projected surface area (Grade GM-10; GraphiteStore, Buffalo Grove, IL, USA). The graphite plates were polished using sandpaper (grit type 400 and 1500), cleaned by soaking in 1 M HCl overnight, and connected to the circuit using titanium wires (5 cm long, 0.08 cm diameter; McMaster-Carr, Cleveland, OH, USA) that pierced the thick rubber stopper used to seal the bottles with an aluminum crimp top. The headspace in the bottles was vacuumed, flushed with an oxygen-free gas mix (CO_2_/N_2_, 20/80) for 10 min, and autoclaved.

Voltage was added to the circuit using a power supply (model 1665; BK Precision, Yorba Linda, CA, USA), with reactors operated in fed-batch mode. Each test lead attached to the positive terminal had a 10 Ω resistor connected in series for recording the voltage produced by each reactor using a multimeter (model 34972A; Agilent Technologies, Santa Clara, CA, USA) in 20 min intervals. A fixed voltage of 0.7 V was applied to all reactors. All reactors were operated at 30°C in the dark without shaking.

AD sludge was collected from a secondary digester at the Pennsylvania State University Wastewater Treatment Plant (University Park, PA, USA). Sediment from a freshwater bog (Black Moshannon State Park, Philipsburg, PA, USA, 40°54 ′20.6″N, 078°03′11.1″W, 20 cm water depth) was placed into glass bottles that were completely filled to minimize oxygen contamination, and then stored at 4°C in the dark. To remove organics it was washed three times using an equal volume of the medium and centrifugation at 13000 × *g* for 20 min and stored at 4°C.

All chemicals were purchased from VWR (Radnor, PA, USA) in the highest available purity. The medium was a phosphate buffered saline (PBS, 100 mM, pH 7.0, containing NaH_2_PO_4_ × H_2_O 9.94 g/L, Na_2_HPO_4_ × H_2_O 5.5 g/L, NH_4_Cl 310 mg/L, KCl 130 mg/L) with 10 mM sodium acetate. The medium was sparged with N_2_ gas for 40 min, autoclaved to remove trace oxygen and cooled down under a flow of N_2_, supplemented with 2.5 g/L (30 mM) NaHCO_3_ (separately sterilized), 5 mL/L of a vitamins solution (mg/L: pyridoxine HCl, 10; thiamin HCl, 5; riboflavin, 5; nicotinic acid, 5; calcium pantothenate, 5; vitamin B_12_, 5; *p*-aminobenzoic acid, 5; thioctic acid, 5; biotin, 2; folic acid, 2; Wolin et al., 1964) and 12.5 mL/L of a minerals solution (g/L: nitrilotriacetic acid, 1.5; MgSO_4_ × 7H_2_O, 3; NaCl, 1; MnSO_4_ × H_2_O, 0.5; NiCl_2_ × 6H_2_O, 0.2; FeSO_4_ × 7H_2_O, 0.1; CoCl_2_, 0.1; CaCl_2_ × 2H_2_O, 0.1; ZnSO_4_, 0.1; CuSO_4_ × 5H_2_O, 0.01; AlK[SO_4_]_2_, 0.01; H_3_BO_3_, 0.01; Na_2_MoO_4_ × 2H_2_O, 0.01). All inocula were prepared in an anaerobic glove box at dilutions of 0.01, 0.1, 1, 10, and 25% (w/v) in PBS medium.

The different inocula (5 mL) were injected into mini-MECs through the stopper using a sterile needle and syringe, leaving 3 mL of headspace. Tests were conducted in triplicates. A batch cycle was considered complete when current dropped below 0.01 mA or methane did not increase. A new cycle was started by injecting 50 μL of a 1 M sodium acetate solution into each reactor. After three cycles (1–2 weeks for each cycle, over a total period of ~1 month) the inoculum-medium mixture was removed from the reactors and 5 mL fresh PBS medium was added. This procedure was repeated until methane production reached stable performance over 3 cycles. Between each cycle, the reactor headspace was vacuumed and purged using CO_2_/N_2_(20/80) for 10 min. Gas production was evaluated at the point of maximum current generation (30 h for AD, 46 h for bog).

### Calculations

Reactor performance was evaluated as previously described (Logan et al., 2008; Wagner et al., 2009), except as noted. Current density was normalized by the anode surface area (mA cm^−2^). Methane production rate was normalized to the reactor liquid volume (mL mL^−1^ cm^−2^) and the anode surface area (1.5 × 2 cm^2^). Methane recovery efficiency (CH_4_ recovery, *CRE* in %), was calculated as the ratio of methane recovered to the maximum possible methane recovery based on the acetate removed (estimated from chemical oxygen demand, COD) and electrical energy input. Coulombic efficiency (*CE* in %) was calculated from Coulombs transferred compared to Coulombs from the substrate removed (COD).

### Gas analysis

Methane concentrations in the headspace were determined using a 250 μL airtight syringe (Hamilton, Reno, NV, USA) and a gas chromatographs (model SRI 310C, 6 foot molecular sieve column in continuous mode at 80°C, SRI Instruments, Torrance, CA, USA).

### Community analysis

Biofilms were dried on the electrodes in a sterile laminar flow hood for about 10 min and then collected by scraping the electrode surface with a sterile scalpel. DNA was extracted using a PowerSoil DNA Isolation Kit (MoBio Laboratories, Inc., Carlsbad, CA, USA) according to the manufacturer's instructions. *Bacteria* and *Archaea* were quantified using qPCR as described previously (Takai and Horikoshi, 2000; Nadkarni et al., 2002). Methanogens were quantified by targeting their *mcrA* genes (Steinberg and Regan, 2009) and *Geobacter* was quantified using an 16S rRNA gene assay (Holmes et al., 2002). To determine the microbial diversity of the two inocula and the reactor electrodes, as well as in the solutions, 16S rRNA genes were sequenced using 454 pyrosequencing with the primers 341F (5′-CCTAYGGGGYGCASCAG-3′) and 1000R (5′-GAGARGWRGTGCATGGCC-3′) for *Archaea* (Gantner et al., 2011) as well as 27F (5′-AGAGTTTGATCCTGGCTCAG-3′; Lane, 1991) and 519R (5′-GTNTTACNGCGGCKGCTG-3′; Ishak et al., 2011) for *Bacteria*. DNA extracts of the reactors with different size inocula were pooled prior to sequencing. Mothur's standard operating procedure for 454 pyrosequencing and a cutoff of at least 300 base pairs was used for data analysis (Schloss et al., 2011). Composite phylogenetic trees of the two inocula were constructed using the arb software package with the SILVA 115 NR99 database using a cutoff of 400 base pairs including redundant sequences to allow a quantitative estimate of clusters found in the samples (Pruesse et al., 2007). With this cutoff, 700 random archaeal and 900 random bacterial sequences were incorporated by the arb maximum parsimony algorithm into the tree. All raw pyrosequencing reads were deposited under the sample accession numbers SRX652342-SRX652357 in the Sequence Read Archive database (Table 1).

**Table 1:** **List of samples with corresponding accession numbers for the Sequence Read Archive http://ncbi.nlm.nih.gov/sra**.

**Sample**	***Archaea***		***Bacteria***	
	**Sample acc. no**.	**Read acc. No**	**Sample acc. no**.	**Read acc. No**
Bog inoulum	SRX652344	SRR1514777	SRX652352	SRR1514785
Anode biofilm bog	SRX652342	SRR1514775	SRX652350	SRR1514783
Cathode biofilm bog	SRX652343	SRR1514776	SRX652351	SRR1514784
Electrolyte using bog	SRX652345	SRR1514778	SRX652353	SRR1514786
AD sludge inoculum	SRX652348	SRR1514781	SRX652356	SRR1514789
Anode biofilm AD sludge	SRX652346	SRR1514779	SRX652354	SRR1514787
Cathode biofilm AD	SRX652347	SRR1514780	SRX652355	SRR1514788
Electrolyte using AD sludge	SRX652349	SRR1514782	SRX652357	SRR1514790

To obtain the Shannon diversity indices, the mothur software package was used. The Shannon index *H* was calculated using:

(1) $$H=-\sum_{i\;=\;1}^{s}p_{i}\ln p_{i}$$

where *s* is the number of species and *p* is the ratio of individuals counted to the total number of individuals (Shannon, 1948). The Shannon index is an indicator of the diversity of the population regardless of the abundance of individual species. A Bray-Curtis similarity coefficient *C* was also calculated using the mothur software, as:

(2) $$C=2\frac{\sum w_{ij}}{\sum a_{i}+\sum b_{j}}$$

where *w* is the lesser value of the common species in the populations A and B, *a* is the number of individual specimens counted in the population A, and *b* is the same number in the population B (Bray and Curtis, 1957). For the Bray-Curtis similarity coefficient a value of 1 means that the two populations are identical and 0 means that they are completely separate. The similarity coefficient *C* is related to the dissimilarity coefficient *D* = 1–C.

## Results

### Methane production

**Figure 1:**
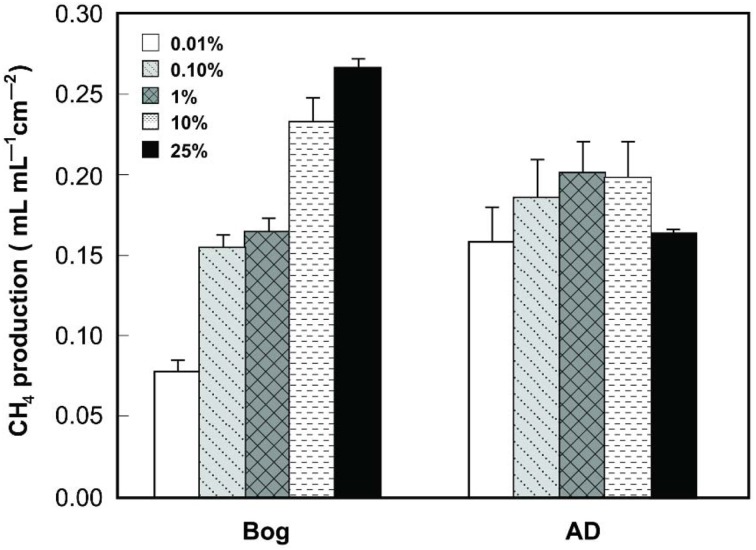
**CH_4_ production (within 40 h) as a function of original inoculum size using either bog or anaerobic digester sludge (AD) inoculum after steady conditions reached after 3 cycles**.

**Figure 2:**
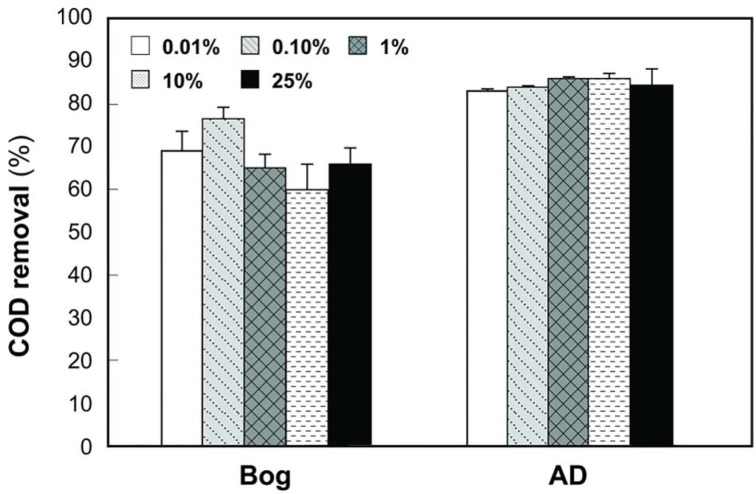
**COD removals as a function of original inoculum size with anaerobic digester (AD) sludge or bog inocula**.

Gas production from the bog samples increased in proportion to the mass of the inoculum, from 0.08 mL mL^−1^ cm^−2^ (0.01% original bog inoculum) to 0.27 mL mL^−1^ cm^−2^ (25%) (Figure 1). In contrast, methane gas production with the AD inoculum was nearly the same for the different inoculum masses, ranging from 0.16 mL mL^−1^ cm^−2^ (0.01%) to a maximum of 0.20 mL mL^−1^ cm^−2^ (1%). COD removal was >80% in all AD tests, but <80% and more variable using the bog inoculum (Figure 2).

### Current generation

**Figure 3:**
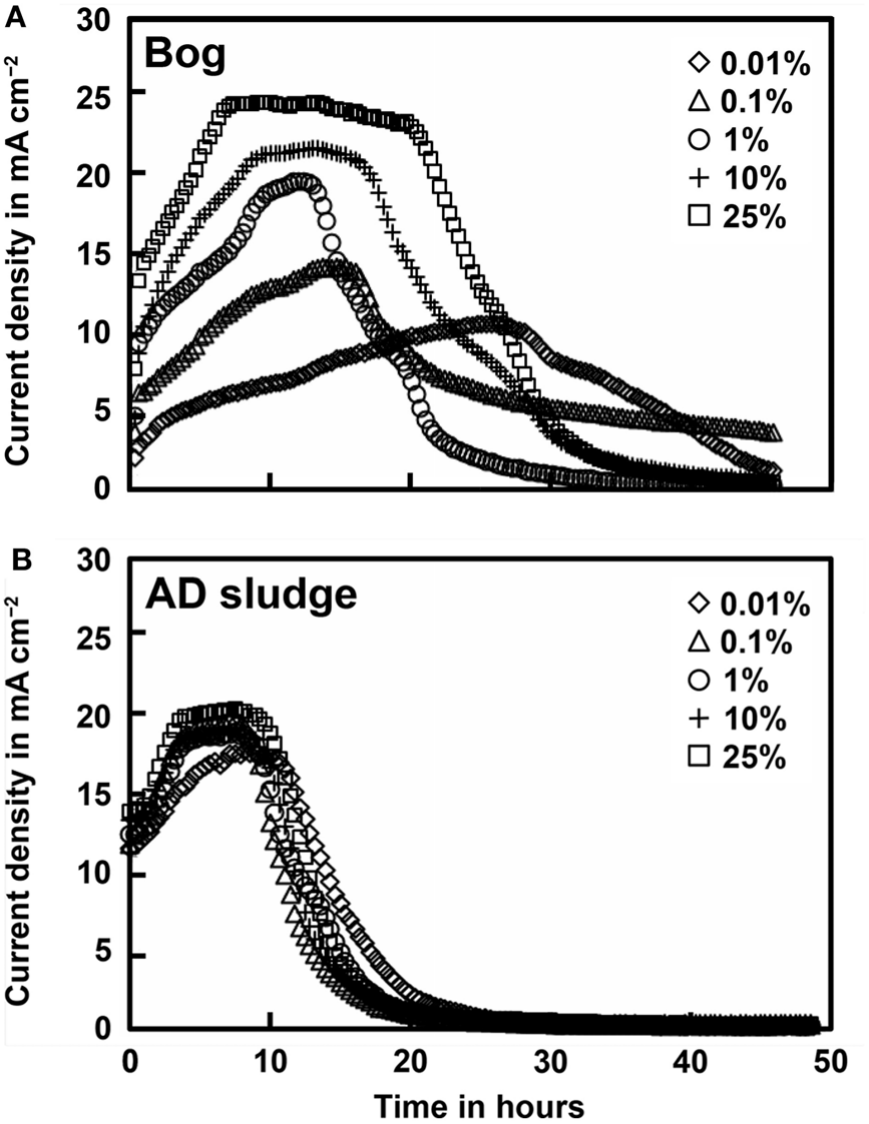
**Current density as a function of original inoculum size of bog (A) and anaerobic digester sludge (B) inoculated reactors after full acclimation of 3 cycles**.

Current densities produced by the bog and AD samples were quite different, but generally showed results consistent with methane generation rates. Peak current densities of the bog samples increased with the inoculum mass, ranging from 0.21 mA cm^−2^ (0.01%) to 0.50 mA cm^−2^ (25%, Figure 3). There were relatively small changes in current densities produced with the different AD inoculum mass. The peak current densities for the AD samples increased slightly from 0.33 mA cm^−2^ (0.01%) to a maximum of 0.42 mA cm^−2^ (25%).

### Coulombic efficiencies and recoveries

**Figure 4:**
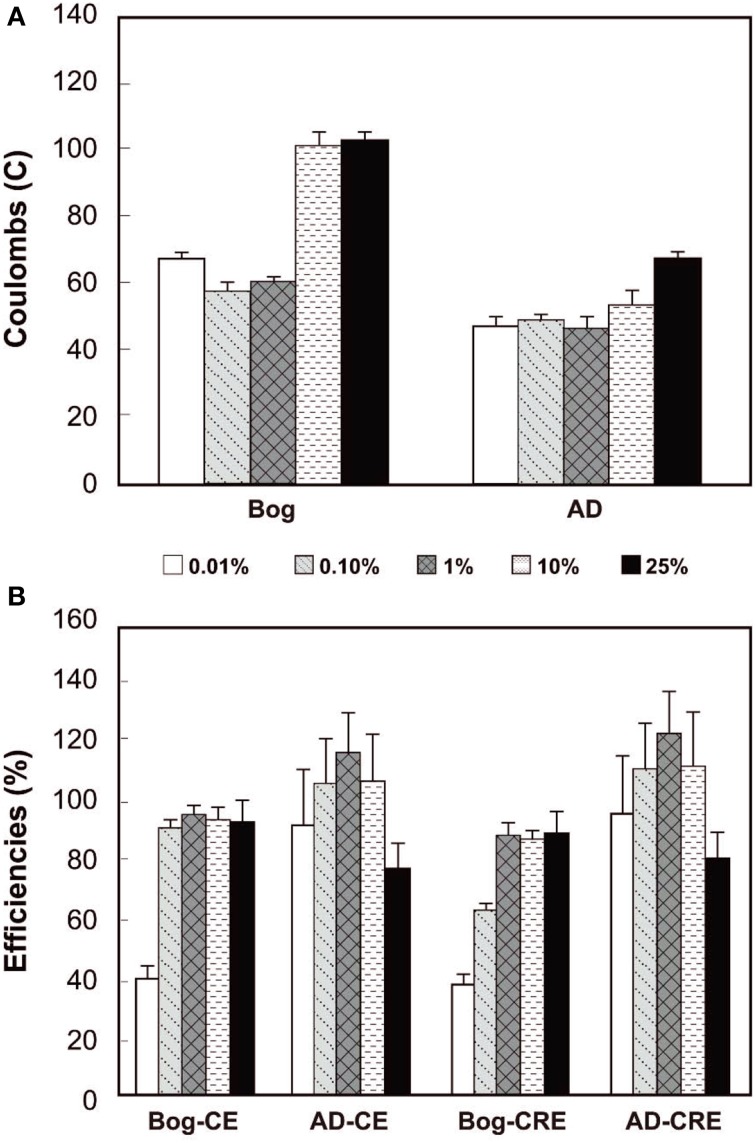
**Coulombs transferred (A) and coulombic efficiency (CE) compared to CH_4_ recovery efficiency (CRE, both B) as a function of original inoculum size with bog and anaerobic digester sludge (AD) after 3 fed-batch cycles**.

The total Coulombs transferred through the circuit is a measure of overall anodic activity, and it is calculated by integrating the current production over time (area under the curves in Figure 3). The largest amount of Coulombs transferred was obtained for the bog sample, with the largest inoculum size (Figure 4). Generally, the total Coulombs transferred in the bog MECs was higher than those in AD ones. With 0.01% inoculum, it was 68 C for bog, and 48 C for AD sludge. The increase was higher for the bog when 25% inoculum was used with 104 C for bog (+53%), but only 69 C for AD sludge (+44%).

The *CE*s reflect the amount of Coulombs transferred compared to the organic matter removed, evaluated on the basis of total COD. The bog inoculum resulted in *CE*s less than 100%, increasing from a *CE* = 39% for the smallest amount (0.01% inoculum) to *CE*s averaging 93 ± 1% for the three largest amounts. The *CE*s for the AD inoculated reactors varied from 91% (0.01% inoculum) to 114% (1% inoculum) with no clear trend with the original inoculum size (Figure 4).

The *CRE* is a measure of total Coulombs recovered in methane compared to Coulombs transferred through the circuit. *CRE*s for the bog samples were all less than 100%, ranging from 38% to 87%. For the AD inoculated reactors, however, some *CRE*s were larger than 100% (e.g., *CRE* = 121% for the 1% inoculum).

### Archaeal communities on the electrodes and in solution

The Shannon diversity index of *Archaea* for the bog inoculum (2.7) was considerably higher than the AD sample (0.3). After 3 batch cycles, the Shannon indices in bog inoculated reactors decreased to 0.7 for the anodes, 0.4 for the solutions and 0.2 for the cathodes. The Shannon indices were slightly higher in the AD inoculated reactors with 1.0 for the anodes, 0.8 for the solutions and 0.6 for the cathodes.

**Figure 5:**
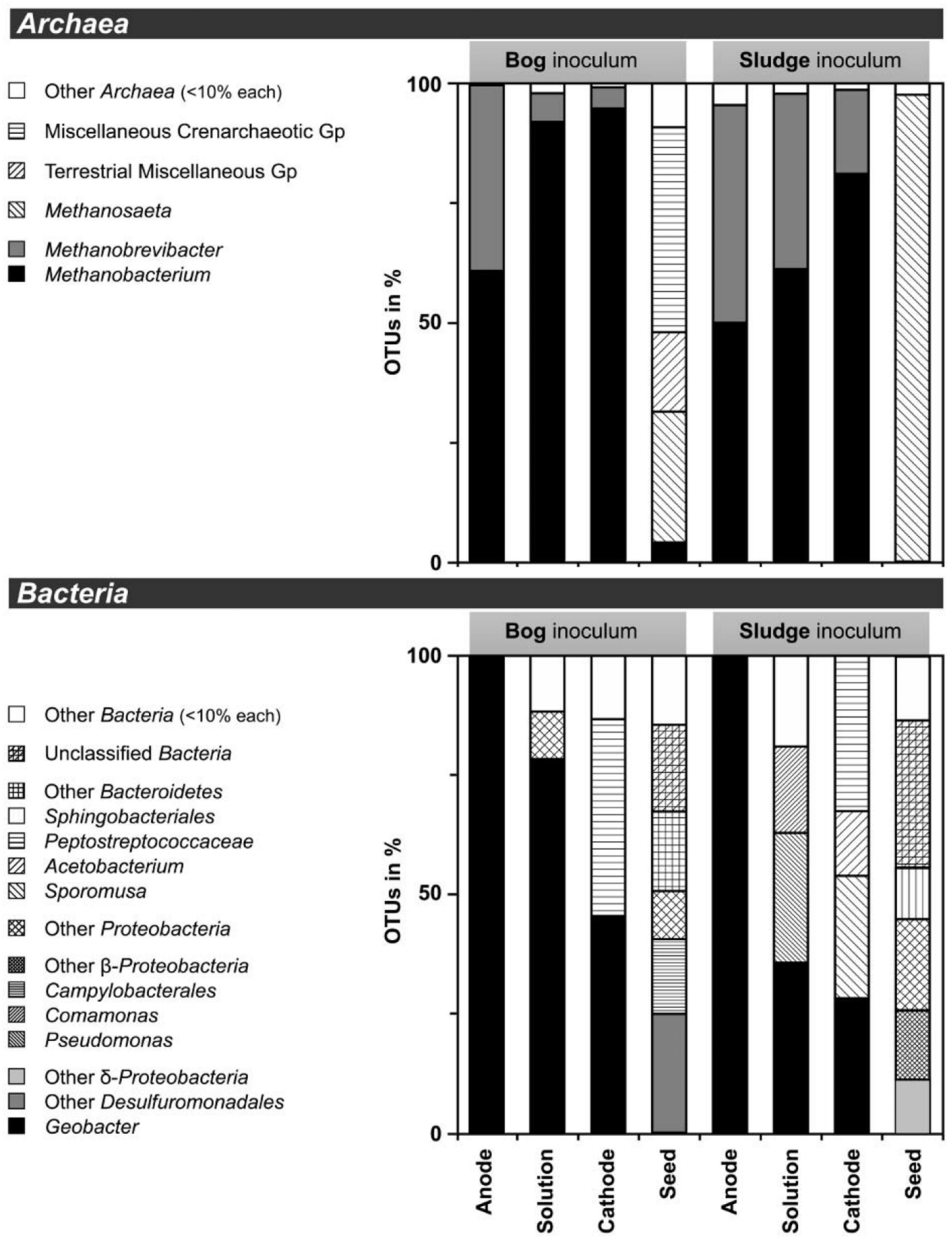
**Distribution of operational taxonomic units (OTUs) of less than 97% sequence similarity in 16S rRNA gene extracted from MEC reactors**. DNA extracts of 0.01, 0.1, 1, 10, and 25% were pooled prior to pyrosequencing. Less than 10% abudant OTUs were summarized as others (white bars). *Archaea* (top) and *Bacteria* (bottom) and were targeted. Reactors were inoculated with bog sediment (left) or AD sludge seed. Gp, group.

**Figure 6:**
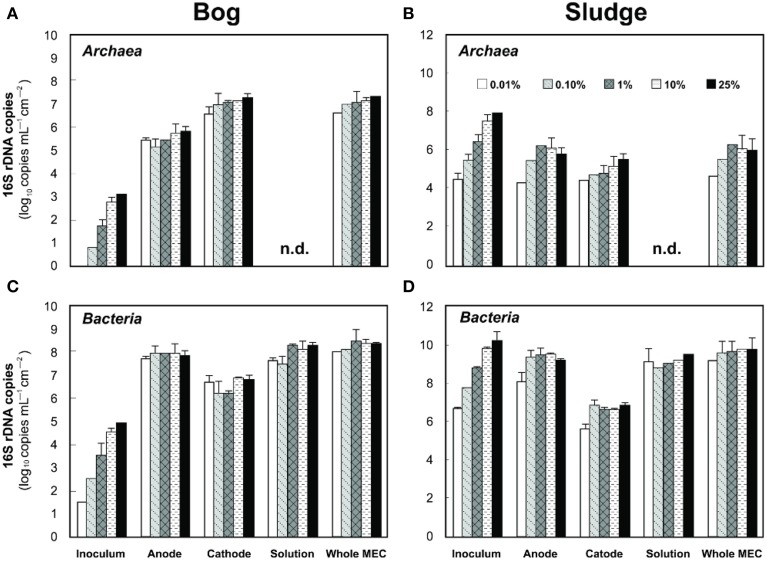
**Left (A,C), copy numbers of *Archaea* (A) and *Bacteria* (C) populations in anodes, cathodes, and solutions of freshwater bog MECs**. Right **(B,D)**, copy numbers of *Archaea* (top) and *Bacteria* (bottom) population in anodes, cathodes and solutions of AD inoculated MECs. n.d., not detected, i.e., below the detection limit of 10^3^ copies.

**Figure 7:**
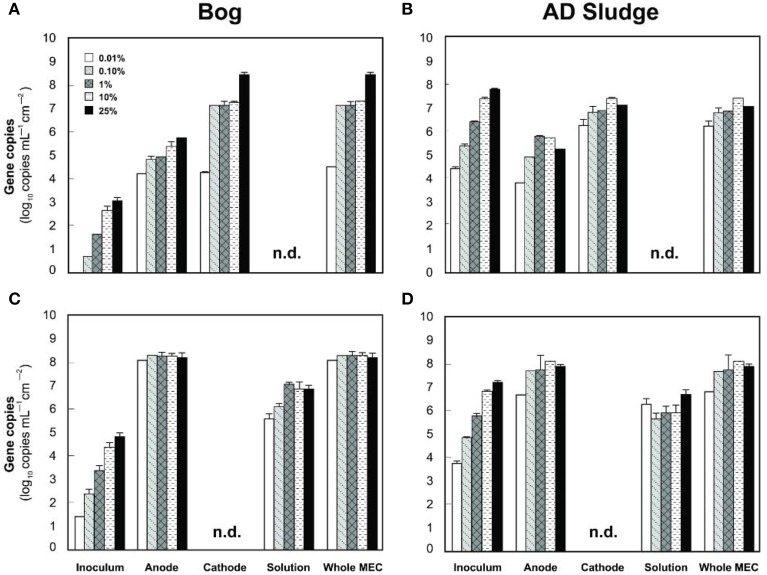
**Copy numbers *mcrA* (A,B) and *Geobacteraceae* (C,D) detected on the anodes, cathodes or solutions of bog (A,C) and AD sludge (B,D) inoculated MECs after 6 fed batch cycles**. An absence of bars shows that copy numbers were below the detection limit (10^3^ copies). Copy numbers of 16 rRNA genes include, but are not restricted to *Geobacteraceae*. n.d., not detected, detection limit 10^3^ copies.

**Figure 8:**
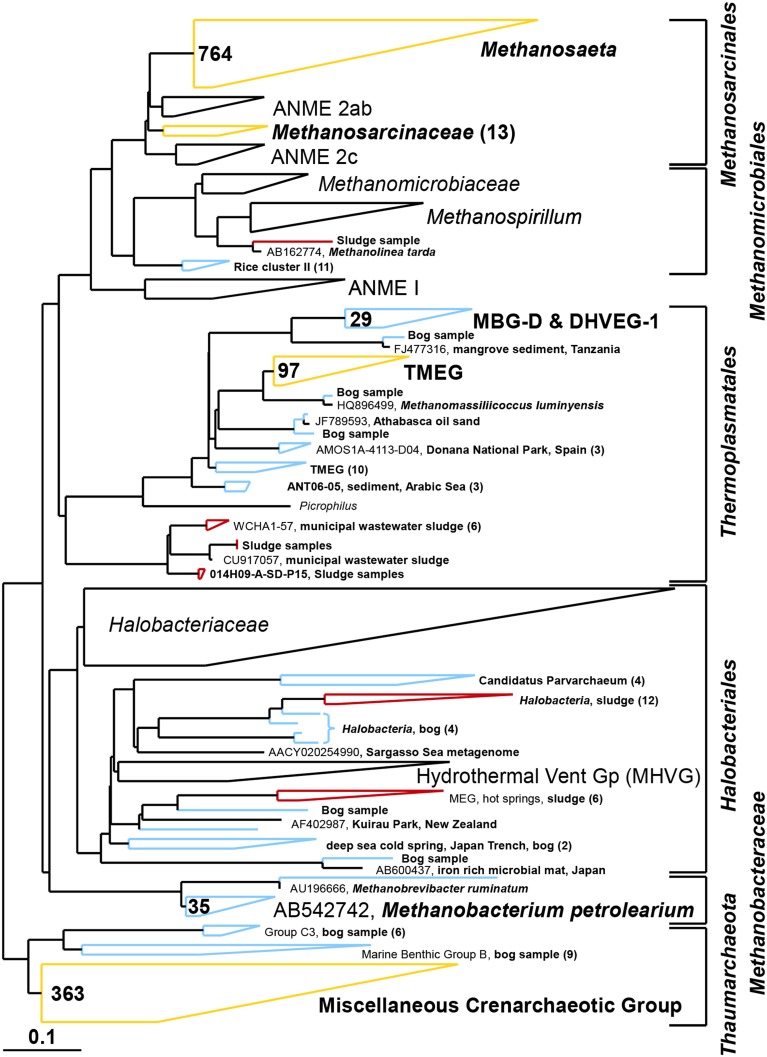
**Composite maximum parsimony phylogenetic tree constructed by incorporating archaeal sequences of the bog sediment (blue) and the AD sludge (red) into an existing SILVA 115 NR99 tree**. Yellow clusters share relatives in both inocula. Numbers indicate total numbers of sequences out of 700 for each sample. No sequences were detected in empty (black) clusters.

Based on pyrosequencing results, the archaeal communities on the cathodes in reactors with either the bog or AD sludge were dominated by microorganisms most similar to *Methanobacterium*, with lesser numbers of *Methanobrevibacter* (Figure 5). There was a slightly increased abundance of *Methanobrevibacter* on the anodes relative to *Methanobacterium*, but overall the communities that evolved over time in these systems were mostly *Methanobacterium*. For all inoculum concentrations, the number of total archaeal cells in the whole reactors (both electrodes plus the medium) determined by qPCR was 2 orders of magnitude higher with the bog inoculum (about 10^6^ for the 0.01% inoculum to 10^7^ copies mL^−1^ cm^−2^ for the 25% inoculum, Figure 6A) than in those seeded with AD sludge (~10^4^ for the 0.01% inoculum to ~10^5^ copies mL^−1^ cm^−2^ for the 25% inoculum, Figure 6B) indicating that the bog sediment was a better inoculum for MEC reactors. Based on these qPCR results, methanogenic *Archaea* comprised about one third of the entire microbial population in the bog reactors after 3 cycles, as opposed to <1% of the microorganisms in the AD sludge reactors (Figures 6, 7). In contrast, the *Archaea*/*Bacteria* ratios in the two inocula were about the same (1/100). However, the archaeal composition of the two inocula was different as indicated by a Bray-Curtis similarity of 0.03 where 1 represents identical samples and 0 completely different samples. *Methanobacterium* and *Methanobrevibacter* species made up about 5% of the *Archaea* in the bog inoculum, while they were below detection limit in the AD sludge (out of 700 sequences, Figure 8).

The analysis of the methanogens in the reactors, based on the number of *mcrA* genes recovered in samples, yielded results similar to those obtained by pyrosequencing. Methanogens in the bog-inoculated reactor were predominantly found on the cathode, except for the 0.01% inoculum where they were equal numbers on both electrodes (Figure 7A). The total number of *mcrA* gene copies recovered from reactors that were inoculated with bog increased from 10^4^ (0.01% inoculum) to 10^8^ mL^−1^ cm^−2^ (25% inoculum), an increase consistent with higher methane production as a function of inoculum size. For the AD sludge inoculated reactors, methanogens were also more abundant (Figure 7B) on the cathode than on the anode, independent of the original inoculum size. In general, final copy numbers of methanogens increased with inoculum mass on the anode to a maximum of 10^5^ copies mL^−1^ cm^−2^ (1% inoculum), and on the cathode to 10^7^ (10% inoculum).

### Bacterial communities on the electrodes

**Figure 9:**
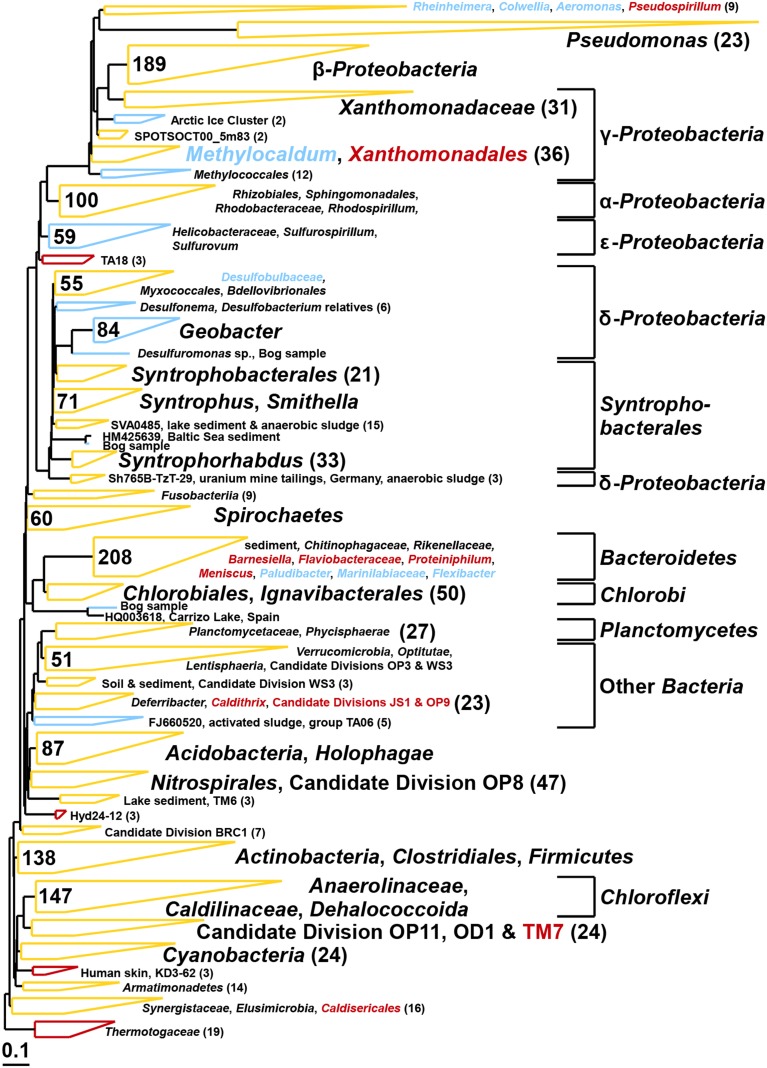
**Composite maximum parsimony phylogenetic tree constructed by incorporating bacterial 16S rRNA gene sequences of the bog sediment (blue) and the AD sludge (red) into an existing SILVA 115 NR99 tree**. Yellow clusters share relatives in both inocula. Numbers indicate total numbers of sequences out of 900 for each sample.

The bacterial communities of both inocula were highly diverse (Shannon diversity index 4.7 for bog and 4.9 for AD sludge) comprising sequences affiliated with α–, β–, γ–, and δ–*Proteobacteria* in both samples (Figure 9). The bog inoculum also contained ε –*Proteobacteria*. Among the δ–*Proteobacteria, Geobacter, Syntrophus*, and *Smithella* species were detected in the bog sample, but not in the AD sludge (out of 900 sequences). Anaerobic ammonium oxidizers (anammox, *Kuenenia*) were only detected in the bog sample. *Chloroflexi* (among them *Anaerolinaceae, Caldilinaceae* and *Dehalococcoida*) and *Bacteroidetes* were observed in both inocula but only the *Bacteroidetes* were different. *Barnesiella, Flaviobacteraceae, Proteiniphilum* and *Meniscus* were discovered only in the AD sludge and *Paludibacter, Marinilabiaceae, Flexibacter* only in the bog sediment. Also *Cyanobacteria* were present in both inocula but *Thermotogaceae* only in the AD sludge. *Pseudomonas* species were observed in both inocula but were more diverse in the bog sediment.

After 3 batch cycles, the genus *Geobacter* dominated the electrodes (Figure 5). *Geobacter* species accounted for 98% of the anode *Bacteria*, while they made up 45% (bog) and 26% (AD sludge) of the cathode *Bacteria*. The only other *Bacteria* in the bog reactors were *Proteobacteria* and *Peptostreptococcaceae*. The Shannon diversity indices decreased to 0.1 for the bog anode, but were higher in the solution with (1.3) and on the cathodes (2.0). Despite the absence of *Geobacter* in the AD inoculum, this genus became dominant over time (98%), resulting in a final Shannon index of 0.2. The solution contained *Pseudomonas* and *Comamonas* species as well, and had a higher Shannon index of 2.0, as did the cathode. Unlike the bog cathode, the AD sludge inoculated reactor had a cathode inhabited by *Acetobacterium* and *Sporomusa*, but both had members of the *Peptostreptococcaceae*.

## Discussion

The performance of the reactors was improved, in terms of current and methane production, by using the bog inoculum. Both inocula resulted in current generation linked to methane production (Figures 1, 3). However, only the AD sludge produced *CE*s greater than 100% (Figure 4). This could be due to hydrogen gas recycling, as has previously been observed in MECs (Rozendal et al., 2008; Lee et al., 2009; Tartakovsky et al., 2009; Rader and Logan, 2010). Hydrogen gas recycling results from oxidation of hydrogen produced at the cathode by microorganisms on the anode, and thus current is produced with no net gas production. *CE*s larger than 100% can also be due to oxidation of solid organic matter or utilization of stored energy in the cells. The observation that *CE*s were larger than 100% for the AD sample, but not the bog sample, suggests that hydrogen gas recycling was occurring in the AD sample due to a lack of efficient conversion of hydrogen gas on the cathode. The rapid conversion of hydrogen to methane by the bog microorganisms may have helped to limit hydrogen gas recycling.

*CRE*s greater than 100% were also observed. Values above 100% could be due to methane production without current generation, which could have been supported by the use of acetate, solid organic matter provided in the inoculum, stored substrates in the cells, or corrosion of the cathode (Siegert et al., 2014). The organic matter added in the AD sample, although washed with medium, was likely much more readily biodegradable than that present in the bog sample, which could have contributed to the large *CRE*s for the AD reactors. However, after 3 cycles all remaining organics from the inoculum were likely degraded. Thus, high *CRE*s were more likely due to corrosion of graphite electrodes. Ultimately, it is not possible to conclusively attribute *CRE*s larger than 100% to any one mechanism as several different mechanisms likely contributed to methane production.

### *Methanobacterium* dominated archaeal communities

It is clear using both inocula that the cathodes were dominated primarily by the genus *Methanobacterium*, as well as to a lesser extent by *Methanobrevibacter* (Figure 5). Apparently, members of these genera which made up about 5–10% of the archaeal bog population were better suited to proliferate using the cathode in the applied voltage MEC conditions than the *Methanosaeta* species which were the only methanogens found in the AD sludge (out of 700 sequences, Figure 5). *Methanobacterium* and *Methanobrevibacter* made up most of the final archaeal population on the cathode in the MEC. The better performance of the bog sample than the AD sludge can be explained by their abundance in the bog inoculum (35 out of 700 sequences, Figure 8). The relative abundance of *Methanobacterium* and *Methanobrevibacter* in these acetate-fed MECs is also consistent with previous reports on archaeal communities in methanogenic biocathodes (Cheng et al., 2009; Sasaki et al., 2011; Van Eerten-Jansen et al., 2013) and iron corrosion (Dinh et al., 2004).

Examination of electrodes and solution using qPCR with *mcrA* primers further supported the conclusion that the bog sediment was better suited for MEC inoculation (Figures 7A,B). It is surprising that more AD sludge inoculum did not result in higher archaeal copy numbers on the cathodes compared with the cell numbers of the inoculum, as seen for the bog sediment (Figures 6, 7). A possible reason could be the use of a graphite electrode material. Graphite, like other carbon based electrodes, is readily colonized by methanogenic communities even under open circuit conditions (Siegert et al., 2014). It seems likely that microorganisms predominant in the AD sludge inoculum would initially adhere to the electrodes, resulting in high colonization of the surface independent of the mass of sludge applied to the surface. Subsequently, there would be less electrode surface available for slower growing *Methanobacterium* and *Methanobrevibacter* species following this initial colonization. Since this start-up condition (negligible *Methanobacterium* and *Methanobrevibacter* compared to *Methanosaeta*) was the same for all AD sludge reactors, but not in the bog reactors (4–10% *Methanobacterium* of all *Archaea* in the inoculum), the final outcome relative to microbial abundance was the same in all AD sludge but not in bog reactors. This hypothesis is also supported by the observation that the Shannon diversity index of the AD reactor doubled from 0.3 in the inoculum to 0.6 on the cathodes. The additional diversity after 3 batch cycles came from the *Methanonbacterium* and *Methanobrevibacter* that were below the detection limit (1 out of 700 sequences) in the AD inoculum. At the same time the diversity index of the bog reactors decreased from 2.7 (inoculum) to 0.2 (cathodes), demonstrating that the remaining archaeal species of the inoculum were not required under operating conditions. Higher numbers of hydrogenotrophic methanogens were found on the cathode than on the anode for both samples (Figure 5), suggesting that hydrogenotrophic or electrotrophic methanogens were predominant in the reactor.

### *Geobacter* dominated anodic bacterial communities

As expected, the anodes in the MECs were dominated by *Geobacter* independent from the inoculum (Figure 5). The good current densities of the MECs here and the prevalence of *Geobacter* on the anodes, are consistent with MFC results where *Geobacter* was essential for good bioelectrochemical reactor performance (Yates et al., 2012). *Geobacter* species were clearly present in the bog inoculum (84 out of 900 sequences, Figure 9), but absent the AD (non-detectable in 900 sequences). Hence, the bog sediment, although containing less than 1% *Geobacter*, was better suited than the AD sludge for inoculating MEC anodes.

The qPCR results confirmed the dominance of the *Geobacter* genus at the anodes. However, the SINA online primer test (Klindworth et al., 2013) showed that the *Geobacter* primers used here (Holmes et al., 2002) also bind to *Desulfuromonadaceae, Desulfobacterales* and *Syntrophobacterales* sequences without mismatches. Since members of these other groups were detected in the AD sludge inoculum and inoculated reactors as well (Figure 5), a quantification of *Geobacteraceae* using this qPCR assay by itself is inconclusive, only supportive of the pyrosequencing results. These results are displayed in the Figures 7C,D.

On the cathodes of reactors inoculated with either inoculum *Peptostreptococcaceae* coexisted with *Geobacter* but could be assigned to *Sporomusa* and *Acetobacterium* only in AD-inoculated reactors (Figure 5). *Sporomusa* is a typical cathodic acetogen (Nevin et al., 2010; Zhang et al., 2013) as well as *Acetobacterium* (Nevin et al., 2011; Marshall et al., 2012). The presence of these two homoacetogenic species could indicate that electrons were not exclusively directed to methanogenesis, and that acetogenesis possibly played a role in methanogen growth. However, the high *CE* of at least 91% is an indicator that most available electrons were converted to methane. The prevalence of hydrogenotrophic methanogens and the high concentrations of acetate added to the reactor make acetate negligible as an intermediate for biocathode methanogenesis.

## Conclusions

These results show that the bog samples were a better inoculum than AD sludge for both improved current generation and methane gas production in MECs. The reason for this was likely due to the relative abundance of *Methanobacterium* and *Geobacter* species in the bog inoculum. The use of the bog inoculum reduced hydrogen gas recycling compared to the AD sample, based on *CE*s that were all less than 100% for the bog sample. On the basis of the different percentages of inoculum used, the AD sample was optimal at 1%, while the bog sample continued to improve in performance up to the maximum of 25%. In all cases, the gas contained methane and no hydrogen gas, indicating either source was effective for methane production.

### Conflict of interest statement

The authors declare that the research was conducted in the absence of any commercial or financial relationships that could be construed as a potential conflict of interest.
